# A comparative study between catalase gene therapy and the cardioprotector monohydroxyethylrutoside (MonoHER) in protecting against doxorubicin-induced cardiotoxicity *in vitro*

**DOI:** 10.1038/sj.bjc.6601430

**Published:** 2003-11-25

**Authors:** M A I Abou El Hassan, M J W E Rabelink, W J F van der Vijgh, A Bast, R C Hoeben

**Affiliations:** 1 Department of Medical Oncology, Free University Medical Center, PO Box 7057, 1007 MB Amsterdam, The Netherlands; 2 Department of Molecular Cell Biology, Leiden University Medical Center, PO Box 9503, 2300 RA Leiden, The Netherlands; 3 Department of Pharmacology and Toxicology, University of Maastricht, PO Box 616, 6200 MD Maastricht, The Netherlands

**Keywords:** doxorubicin, MonoHER, human catalase, adenovirus, neonatal cardiac myocytes, MTT

## Abstract

Cardiotoxicity is the main dose-limiting side effect of doxorubicin in the clinic. Being a free radical producer, doxorubicin affects the heart specifically because of its low antioxidant capacity. Among those antioxidants, catalase is present in very low levels in the heart compared to other organs. Since catalase is an essential enzyme in detoxifying hydrogen peroxide, the aim of the present study was to investigate the protective effect of catalase as delivered by an adenovirus vector against doxorubicin-induced cardiotoxicity in cultured neonatal rat cardiac myocytes (NeRCaMs). 7-Monohydroxyethylrutoside (MonoHER), a potent cardioprotector currently under clinical investigations, was included in the study as a reference. Neonatal rat cardiac myocytes were infected with different multiplicity of infections (MOIs) of adenovirus encoding catalase (AdCat). A control infection with an adenovirus vector encoding a nonrelated protein was included. The activity and content of catalase in infected cells were determined during 3 days postinfection. One group of NeRCaMs was infected with AdCat before treatment with doxorubicin (0–50 *μ*M). The second and third group were treated with doxorubicin (0–50 *μ*M) with and without 1 mM monohydroxyethylrutoside (monoHER), respectively. The LDH release and viability of treated cells were measured 24 and 48 h after doxorubicin treatment. The beating rate was followed in three other groups of cells receiving the same treatments within 3 days after doxorubicin (0–100 *μ*M) treatment. Catalase activity increased in AdCat-infected cells, with different MOIs, starting from the second day after infection as compared to the mock-infected cells (*P*<0.03). At the third day of infection, an MOI of more than 50 caused cytopathic effects, which hampered the use of higher viral titres. With an MOI of 50, catalase activity increased 3.5-fold in AdCat-infected cells 3 days postinfection (*P*=0.021) compared to mock-infected cells. The beating rate and survival of NeRCaMs decreased in a concentration and time-dependent manner after doxorubicin treatment (*P*<0.0005). This cytotoxicity was associated with an increase in the LDH release from the treated cells (*P*<0.0005). The cells stopped beating 24 h after treatment with >50 *μ*M doxorubicin. A 3.5-fold increase in the activity of catalase did not protect NeRCaMs against any of the cytotoxic effects of doxorubicin on NeRCaMs. In contrast, monoHER (1 mM) significantly protected NeRCaMs against the lethal effects of doxorubicin on the survival, LDH release and the beating rate of NeRCaMs (*P*<0.004) during 48 h after doxorubicin treatment. This protection resulted in a prolongation of the beating of doxorubicin-treated cells after the end of the experiment (i.e. >72 h). The present study (1) illustrates that the cytotoxicity of high MOI of AdCat (>50) limited the possibility to increase catalase activity more than 3.5-fold, which was not enough to protect infected NeRCaMs against doxorubicin-induced cardiotoxicity and (2) confirms the efficacy of monoHER as a cardioprotector. Thus, the use of monoHER proves more suitable for the prevention of doxorubicin-induced cardiotoxicity than catalase gene transfer employing adenovirus vectors.

Free radicals are considered the fundamental mediators of doxorubicin-induced cardiotoxicity. The semiquinone form, produced by the reduction of doxorubicin, interacts with molecular oxygen yielding superoxide radicals (Goeptar *et al*, 1993). These radicals are rapidly transformed either spontaneously or enzymatically into hydrogen peroxide (H_2_O_2_), which is converted into hydroxy radicals (OH^•^) (Kalyanaraman *et al*, 1984). These highly reactive species interact specifically with heart tissue because of its low antioxidant content.

Several studies were conducted using exogenous metal ion chelators and/or antioxidants like ICRF-187 and 7-monohydroxyethylrutoside (monoHER). These studies seemed promising in the protection against doxorubicin-induced cardiotoxicity *in vitro* and *in vivo* (Van Acker SABE *et al*, 1997; Van Acker FAA *et al*, 2000; Koning *et al*, 1991) without interfering with its antitumour activity. ICRF-187, however, showed transient leucopenia and moderate thrombocytopenia (Koning *et al*, 1991) and thus amplified the myelotoxicity of doxorubicin. The cardioprotection of monoHER was attributed to both potent free radical scavenging and metal ion chelating properties (Van Acker *et al*, 1996, 1998) of the compound and thus compensating for the low antioxidant content of the heart tissue (Green *et al*, 1990; Keizer *et al*, 1990; Lin *et al*, 1991; Takacs *et al*, 1992). 7-Monohydroxyethylrutoside is under early clinical investigation, and the search for other cardioprotectors is therefore warranted.

Catalase is a major enzyme involved in the detoxification of H_2_O_2_ into water and molecular oxygen (James Kang *et al*, 1996). Catalase activity is high in the liver and erythrocytes, but very low in the heart of man (Ishikawa *et al*, 1986) and mice (Doroshow *et al*, 1980).

The use of exogenous catalase in the protection against oxidative stress proved effective *in vitro* (Kirshenbaum and Singal, 1993; Thayer, 1986), but it showed contradictory results *in vivo* because of catalase instability or reduced ability to penetrate cells (Villani *et al*, 1992).

The endogenous production of high levels of catalase in transgenic mice definitely proved the efficacy of catalase in the protection against oxidative damage as produced by ischaemia reperfusion (IR) (Li *et al*, 1997) and doxorubicin (James Kang *et al*, 1996). These encouraging results prompted us to explore the protective effect of catalase gene therapy against doxorubicin-induced cardiotoxicity.

The aim of the present study was to investigate the protective effect of adenoviral-cardiac gene transfer of catalase against the cytotoxic effects of doxorubicin on the survival, LDH release and the beating rate of Neonatal rat cardiac myocytes (NeRCaMs) *in vitro*. The cardioprotector monoHER was used as a reference in the present investigation.

## MATERIALS AND METHODS

### Adenovirus vectors

The catalase encoding adenovirus (AdCat) (Danel *et al*, 1998) (was kindly provided by Dr RG Crystal, Joan and Sanford I Weill Medical College, Cornell University) contains the cDNA of human catalase driven by the Ad2 major late promoter (MLP). The vector was generated by the ligation of the ClaI cut plasmid pPB369 with the large ClaI fragment of the Ad5 E3 deletion mutant Addl327 (Erzurum *et al*, 1993). An adenovirus encoding nonrelated protein was used for control infection. The adenovirus vectors were propagated in PER C6 cells (Fallaux *et al*, 1998) and purified by CsCl density gradient. The viral preparations were dialysed and stored at –80°C until use. The titre of each viral stock was determined on 911 cells (Fallaux *et al*, 1996). The titres ranged between 4–5 × 10^10^ plaque-forming unit (p.f.u.) ml^−1^. The adenovirus stocks were free of replication competent adenovirus (RCA) as tested by PCR using primers flanking the AdE1A region (Pietersen *et al*, 1999).

### Neonatal rat cardiac myocytes isolation and culture

Hearts were aseptically removed from 16 neonatal Sprague–Dawly rats (2–3 days old from Harlan Nederland, Horst, The Netherlands) after decapitation. After removal of the atria, the ventricles were carefully dissected and washed with a buffer solution (pH 7.3), which contained NaCl (137 mM), KCl (5.4 mM), Na_2_HPO_4_ (0.34 mM), KH_2_PO_4_ (0.44 mM), D-glucose (5.56 mM), 4-(2-hydroxyethyl)-1-piperazineethanesulphonic acid (HEPES) (20 mM) and 0.02% phenol red. The dissected tissue was digested with collagenase-deoxyribonuclease solution containing 14 U ml^−1^ collagenase (Worthington Biochemical Corporation, Freehold, NJ, USA), 450 U ml^−1^ deoxyribonuclease (DNase, Worthington Biochemical Corporation), 10 *μ*M CaCl_2_ and 0.6 mM MgCl_2_ (Van Wamel *et al*, 2000).

The digestion mixture was incubated at 37°C for 20 min by shaking in the presence of glass beads, which assists the release of free cells from the digested tissue. The digestion step was repeated for undissociated tissue, and the cell suspension of the two digestion steps was pooled in one tube kept on ice. The cells were spun down by centrifugation at 240 g for 15 min. The cell pellet was suspended in preplating medium – containing Ham's F-10 (Life Technologies, Breda, The Netherlands), 10% fetal bovine serum (FBS, Life Technologies) and 10% horse serum (HS, Life Technologies) – and preplated in a 10 cm Petri dish (Falcon Primaria, Becton-Dickinson, Etten-Leur, The Netherlands) at 37°C. The fibroblasts were left to attach for 25 min.

The unattached NeRCaMs were collected by careful shaking of the plates. The myocyte cell suspension was divided over six- or 24-well plates with a final cell density of 5 × 10^4^ cell cm^−2^. Neonatal rat cardiac myocytes were left to attach for 5–7 h. Thereafter, the preplating medium was refreshed with culture medium consisting of equal portions of Dulbecco's modified Eagle's medium (DMEM, Life Technologies) and Ham's F-10 and containing 100 U ml^−1^ penicillin, 100 *μ*g ml^−1^ streptomycin and 5% HS. Cells were incubated at 37°C in humidified air containing 5% CO_2_. These cultures contained at least 90% of synchronously beating NeRCaMs. The medium was refreshed after 24 h. At 48 h thereafter, NeRCaMs cultures were ready for use.

### *In vitro* gene transfer and catalase activity and content determination

Neonatal rat cardiac myocytes cultured in six-well plates were incubated with different MOIs (25, 50 and 75) of AdCat in growth medium (1 ml well^−1^) at 37°C. At 2 h postinfection, another volume of virus-free growth medium was added. Control cells were infected with adenovirus encoding an unrelated protein (MOI 75).

The cells were lysed 24, 48 and 72 h postinfection with Ripa buffer (50 mM tris-HCl, 150 mM NaCl, 0.1% SDS, 0.5% sodium deoxycholate (DOC, Fluka Biochemika, Buchs, Switzerland) and 1% nonidet P40 (NP40, Fluka Biochemika)). The cellular lysates were immediately placed on ice for catalase activity determination and the remaining lysates were kept at –80°C for Western blot analysis.

The catalase activity was measured by the H_2_O_2_ degradation assay (Aebi, 1984). In brief, 30 *μ*l of cellular lysate was mixed with 1 ml of TPH buffer (containing 0.02% triton X-100 (Backer, Deventer, The Netherlands) and 0.03% H_2_O_2_ (Backer) in phosphate buffer (50 mM, pH 7.0)) in a quartz cuvette. The decrease in absorbance (H_2_O_2_ degradation) was followed at a wavelength of 240 nm for 30 s. The catalase activity was calculated by interpolating the rate of H_2_O_2_ degradation (Δ*A* min^−1^) of each test sample on the calibration curve obtained by plotting the Δ*A* min^−1^ against the catalase activity of the calibration samples (freshly prepared on the day of analysis).

For the determination of the catalase content, 50 *μ*g protein of each sample (determined by Biorad total protein assay (Bio-Rad Laboratories B.V., Veenendaal, The Netherlands) was separated on a 12.5% SDS–PAGE. Thereafter, the proteins were blotted on Immobilon membrane (Millipore B.V., Etten-Leur, The Netherlands). The membrane was rinsed in blocking solution containing 5% nonfat milk, 0.2% Tween 20, 150 mM NaCl and 10 mM Tris-HCl, pH 8. Subsequently, catalase protein was immunoprobed with sheep anti-human catalase 1^ry^antibody (1 : 500 dilution) (The Binding Site, Birmingham, UK). The blot was incubated with peroxidase-conjugated donkey anti-sheep IgG 2^ry^ antibody (Jackson Immuno Research Laboratories Incorporation, West Grove, USA) (1 : 1000 dilution). For the chemiluminescence detection, the blot was immersed in 0.025% luminol solution containing 0.01% H_2_O_2_ and exposed to hyperfilm (Kodak London, UK).

### MTT (3-(4,-dimethylthiazol-2-yl)-2,5-diphenyltetrazolium bromide) assay and LDH release

**Figure 1:**
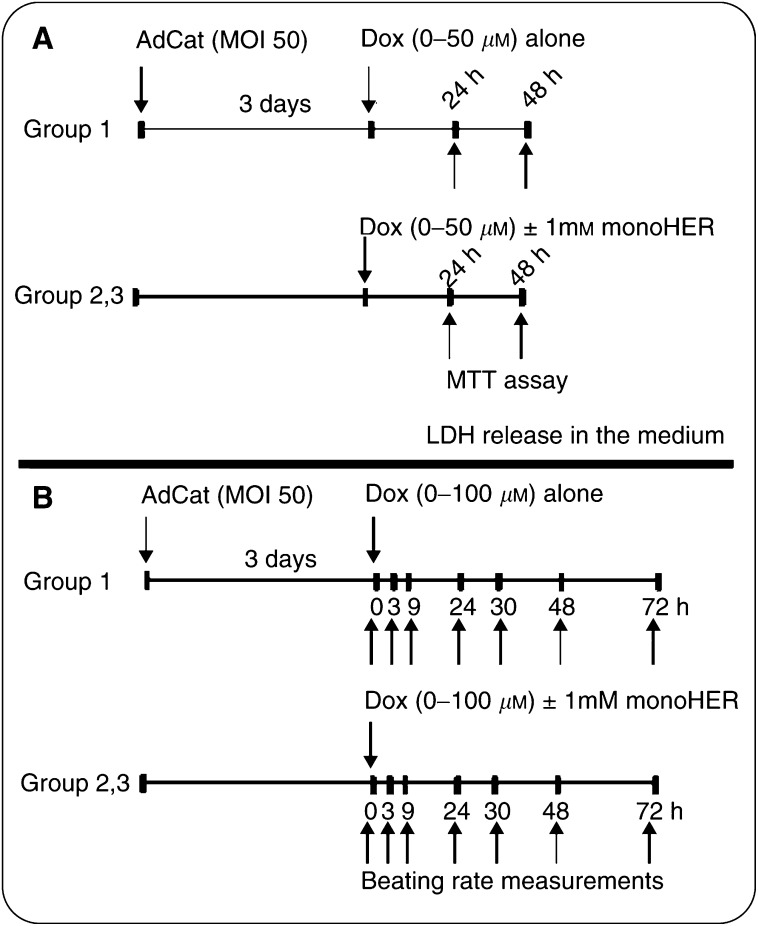
Schematic presentation of the study design.

The cells grown in 24-well plates were divided into three groups (see study design in Figure 1A

). In one group the cells were infected with AdCat. At 3 days postinfection, the cells were treated with different concentrations of doxorubicin (0–50 *μ*M). In parallel, the other two groups of noninfected NeRCaMs were treated with doxorubicin (0–50 *μ*M) alone or with 1 mM monoHER (solubilised with 0.5% DMSO). In all, 0.5% of DMSO did not show any effect on the viability or LDH release from treated cells compared to the blank. At 24 and 48 h after doxorubicin treatment, the medium was collected for LDH measurement and the cells were washed with phosphate-buffered saline for the MTT assay.

The LDH activity was measured (Moldeus *et al*, 1978) by mixing 10 *μ*l of the medium with 10 *μ*l of a 50 mM sodium pyruvate (Merck, Amsterdam, The Netherlands) solution in 1 ml TEA buffer (containing 500 mM triethanolamine, 100 mM MgCl_2_, 50 mM EDTA and 0.2 mM NADH (Sigma Aldrich Chemie, Zwijndrecht, The Netherlands)). The rate of NADH oxidation was followed at a wavelength of 340 nm. The percentage LDH release in the medium was plotted against the doxorubicin concentration (taking the blank as 0% release).

The MTT assay was performed according to Mosmann (1983) with minor modifications. The percentage survival (taking the blank as 100% survival) was plotted as a function of the doxorubicin concentration.

### Beating rate measurement

Three groups of cells were assigned for the beating rate measurement (see study design in Figure 1B). The first group was infected with AdCat followed by doxorubicin treatment (0–100 *μ*M) 3 days postinfection. The other groups were treated with different concentrations of doxorubicin (0–100 μM) with or without 1 mM monoHER (solubilised with 0.5% DMSO). In all, 0.5% DMSO alone did not affect the beating rate of treated cells compared to the blank. The mean beating rate of treated cells was manually counted in two fixed spots per well within 72 h after doxorubicin treatment. During counting, the CO_2_ atmosphere and temperature around the cells were kept comparable to that in the incubator by sealing the plates with parafilm and placing them on a transparent thermostat box (39°C) placed over the microscope's stage. The temperature in the medium was about 34°C as calibrated before the start of the experiment. Under these conditions, the beating rate was stable for at least 30 min.

In order to validate the manual counts, the beating of the cells was recorded on sVHS tapes through a phase contrast inverted microscope connected to a video camera. Sequences of beating images were saved as AVI-files using the Studio DC10plus program (Pinnacle Systems) with a frame rate of 25 Hz. A computer program was developed for the analysis of beating images and the calculation of the beating rate of the moving NeRCaMs.

The percentage of beating rate (the value of the beating rate of treated cells at a certain time × 100/the beating rate of the same cells at zero time) was plotted against time (h).

### Statistical analysis

The increase in the total catalase activity in NeRCaMs infected with AdCat (different MOIs) was statistically evaluated by ANOVA for the multiple regression of catalase activity on AdCat MOI and days after infection. In addition, Student's *t*-test was used to evaluate the activity of catalase in cells infected with different MOIs of AdCat compared with the blank or the control infection at the respective day.

The change in the LDH release or survival of NeRCaMs treated with different concentrations of doxorubicin alone, with 1 mM monoHER or with AdCat infection was statistically evaluated by the ANOVA for multiple regression of ln (viability or LDH release) on ln doxorubicin concentration alone, with 1 mM monoHER or with AdCat (MOI 25) infection. For the beating rate, the change was evaluated by the ANOVA for the multiple regression of ln % beating rate on ln time for each concentration of doxorubicin alone, with monoHER or with AdCat preinfection.

## RESULTS

### Catalase activity and content

**Figure 2:**
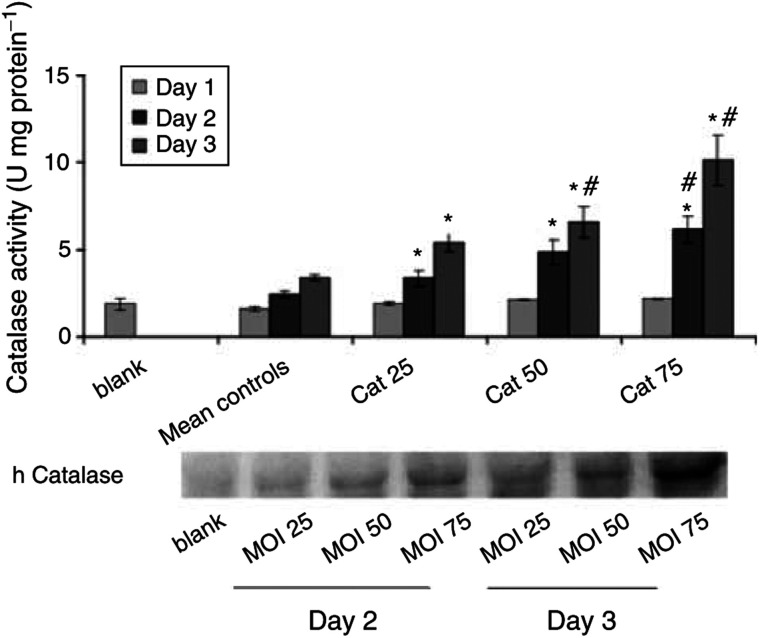
Activity and content of catalase in NeRCaMs within 3 days postinfection with different MOI of AdCat. Values are mean (*n*=3) ± s.e.m. The *P*-values (^*^ compared to blank *P*< 0.03 and ♯ compared to the control *P*< 0.05), which indicate significant increase in the catalase activity, were calculated by Student's *t*-test.

The activity and content of catalase in NeRCaMs infected with different MOIs (25, 50 and 75) of AdCat are shown in Figure 2

. No significant increase in the catalase activity in NeRCaMs was observed after control infection compared to untreated cells (blank, *P*<0.485) up to 72 h postinfection. Catalase activity significantly increased at the third day of AdCat infection (with different MOIs) compared to the blank (*P*<0.03) and to the control (*P*<0.05). No significant difference was observed between the catalase activity in NeRCaMs infected with different MOIs of AdCat 3 days postinfection. Cytopathic effects (CPE, cell rounding up and increased acidity of the medium) limited the use of an MOI of >50 at the third day postinfection.

The Western blot of catalase confirms the activity measurements and specifically shows a considerable MOI- and time-dependent increase in the expression of catalase in cells infected with different MOIs of AdCat within 3 days postinfection.

### Survival and LDH release

Figures 3

**Figure 3:**
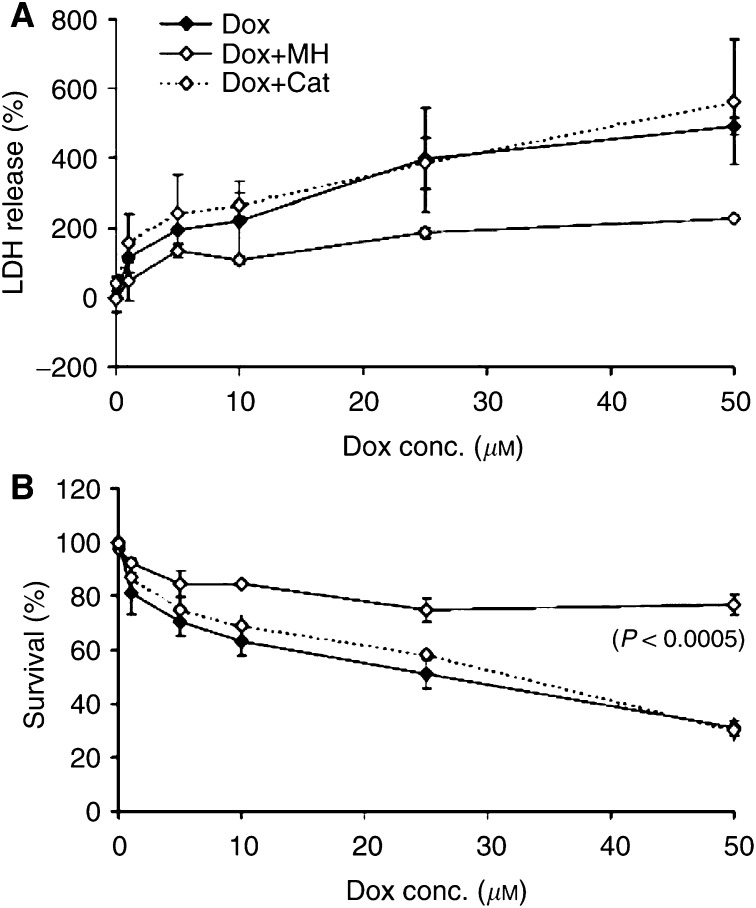
Protective effect of preinfection with AdCat (MOI 50) or cotreatment with 1 mM monoHER against doxorubicin-induced LDH release (**A**) and survival (**B**) of NeRCaMs 1 day after doxorubicin treatment. Values are mean (*n*=3) ± s.e.m. The *P*-values, which indicate significant protection against doxorubicin damage, were calculated by the ANOVA of multiple regression of ln (survival or LDH release) *vs* ln doxorubicin concentration alone, with 1 mM monoHER or with AdCat (MOI 50) preinfection.

and 4

**Figure 4:**
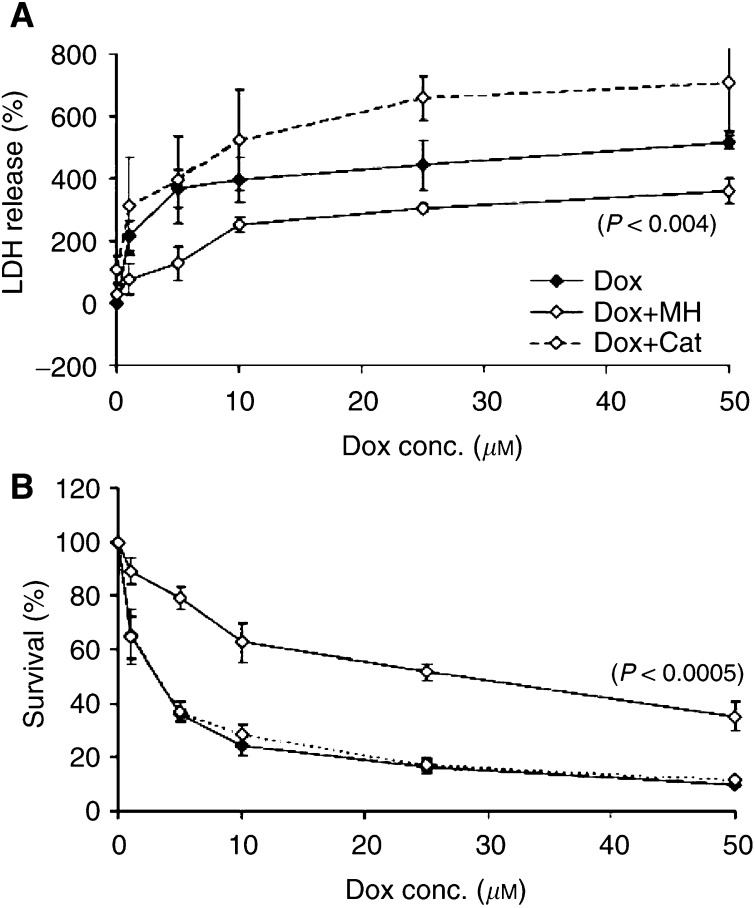
Protective effect of preinfection with AdCat (MOI 50) or cotreatment with 1 mM monoHER against doxorubicin-induced LDH release (**A**) and survival (**B**) of NeRCaMs 2 days after doxorubicin treatment. Values are mean (*n*=3) ± s.e.m. The *P*-values, which indicate significant protection against doxorubicin damage, were calculated by the ANOVA of multiple regression of ln (survival or LDH release) *vs* ln doxorubicin concentration alone, with 1 mM monoHER or with AdCat (MOI 50) preinfection.

show the survival of NeRCaMs and the LDH release 24 and 48 h after treatment with different concentrations of doxorubicin with or without preinfection with AdCat (MOI 50) or cotreatment with 1 mM monoHER (MH). Doxorubicin caused cytotoxic effects in NeRCaMs. A significant concentration-dependent reduction in the percentage of surviving NeRCaMs was observed within 48 h after treatment (*P*<0.0005). Cell death was, as expected, accompanied by a significant increase in the LDH release in the medium of doxorubicin-treated cells (*P*<0.0005).

No cytotoxic effects (reduced viability or increased LDH release) were observed after infection with AdCat (MOI 50) during 5 days after infection. A 3.5-fold increase in the activity of AdCat did not protect against doxorubicin-induced reduction in the survival of NeRCaMs or the associated increase in the LDH release from the myocytes within 48 h after doxorubicin treatment.

On the other hand, 1 mM monoHER alone had no influence on the viability or LDH release from treated NeRCaMs during 48 h after treatment. 7-Monohydroxyethylrutoside significantly protected against the lethal effect of doxorubicin on the survival of NeRCaMs during 48 h after doxorubicin treatment (*P*<0.0005). 7-Monohydroxyethylrutoside also significantly decreased doxorubicin-induced LDH release from NeRCaMs 48 h after doxorubicin treatment (*P*=0.004).

### Beating rate

**Figure 5:**
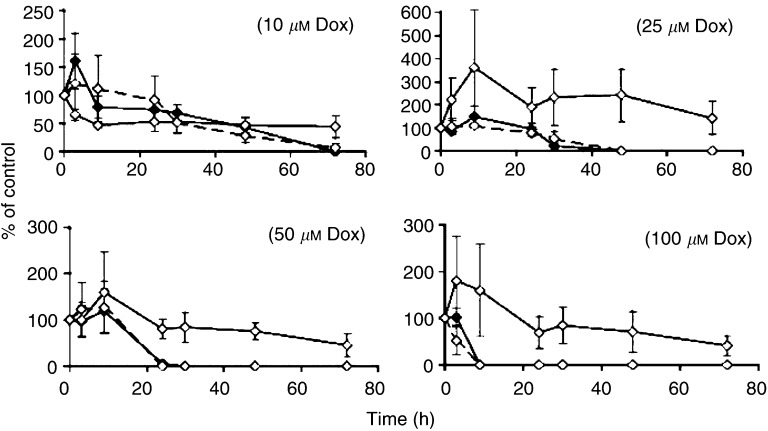
Beating rate of NeRCaMs within 3 days of treatment with different concentrations of doxorubicin (–♦–) with(out) preinfection with AdCat (MOI 50) (⋯◊⋯) or cotreatment with 1 mM monoHER (–◊–). The *P*-values, which indicate significant protection against doxorubicin effect, were calculated by the ANOVA of multiple regression of ln (beating rate) *vs* ln doxorubicin concentration alone, with 1 mM monoHER or with AdCat (MOI 50) preinfection.

The beating rate of NeRCaMs within 3 days after treatment with different concentrations of doxorubicin with or without preinfection with AdCat (MOI 50) or cotreatment with 1 mM monoHER is shown in Figure 5

. The beating rate of untreated cells (blank) was relatively stable during the course of the experiment and was 109±23 bpm. Infection with AdCat (MOI 50) or treatment with 1 mM monoHER alone did not significantly influence the beating rate of NeRCaMs compared to the blank.

Doxorubicin significantly decreased the beating rate of NeRCaMs in a concentration- and time-dependent manner (*P*<0.0005). The time at which cells stopped beating (*t*_zero_) after different treatments is summarised in Table 1

**Table 1:** *T*_zero_ of NeRCaMs treated with different concentrations of doxorubicin (10–100 *μ*M) with or without 1 mM monoHER or preinfection with AdCat (MOI=50). Values are mean (*n*=3) ±s.e.m.

**Dox**	***T*_zero_ (h)**		
***μ*M**	**Dox**	**Dox + AdCat**	**Dox + MH**
**10**	72.0±0.0	72.0±0.0	>72
**25**	42.0±6.0	48.0±0.0	>72
**50**	26.0±2.0	24.0±0.0	>72
**100**	9.0±0.0	7.0±2.0	>72

. The *t*_zero_ was inversely proportional to the doxorubicin concentration and ranged from 72 to 9 h after treatment with 10 and 100 *μ*M doxorubicin, respectively.

Infection with AdCat (MOI 50) did not protect against the negative chronotropic effect of doxorubicin on NeRCaMs during 72 h after doxorubicin treatment (Table 1, Figure 5). Cotreatment with 1 mM monoHER significantly protected against doxorubicin-induced reduction of the beating rate of NeRCaMs (*P*<0.0005). 7-Monohydroxyethylrutoside prolonged the *t*_zero_ for >72 h even in the presence of high concentrations (50, 100 *μ*M) of doxorubicin (Table 1, Figure 5).

## DISCUSSION

Enhancing the low antioxidant capacity of heart tissue was the rationale behind several studies conducted for the protection against doxorubicin-induced cardiotoxicity (Keizer *et al*, 1990). This cardiotoxicity has mainly been attributed to the production of free radicals. Doxorubicin can generate free radicals in different ways. It can be enzymatically and nonenzymatically reduced to the semiquinone form (Goeptar *et al*, 1993, 1994). In the presence of oxygen, the semiquinone form donates the extra electron to molecular oxygen producing superoxide anion radical (O_2_^•−^). Spontaneous or enzymatic dismutation of two molecules of O_2_^•−^ produces H_2_O_2_. Hydrogen peroxide can also be produced by redox cycling of doxorubicin after complexation with iron cations (Sterrenberg *et al*, 1984; Minotti, 1989). Hydrogen peroxide can be converted into hydroxyl radicals, through Fenton reaction (Yu and Anderson, 1997), which are much more toxic than O_2_^•−^.

In a previous study using a superoxide dismutase (CuZn-sod)-encoding adenovirus (AdCuZn-sod), we have shown that moderate overexpression of CuZn-sod did not protect against doxorubicin-induced cardiotoxicity in cultured cardiac myocytes (Abou El Hassan, 2003).

Even more, the AdCuZn-sod induced cytotoxicity at a relatively low MOI of more than 25 virus infectious units per cell. This cytotoxicity might be caused by the viral vector or by CuZn-sod itself because its conversion of the doxorubicin-produced O_2_^•−^ into H_2_O_2_ in the presence of low levels of catalase would cause accumulation of H_2_O_2_, which is subsequently converted into hydroxyl radicals, which are much more reactive than O_2_^•−^ radicals in inducing cellular damage. This implies that the overexpression of CuZn-sod in cells harbouring low levels of catalase, such as cardiac myocytes, would cause damage rather than protection of these cells, which is supported by the data of Xing *et al* (2002) and Midorikawa and Kawanishi (2001).

Catalase is responsible for converting H_2_O_2_ to water and oxygen (Deshmukh *et al*, 1997). The level of catalase in heart tissue presents only 2% of that in the liver in different species (Doroshow *et al*, 1980; Ishikawa *et al*, 1986; Chen *et al*, 1994), which allows the conversion of undetoxified H_2_O_2_ into hydroxy radicals (OH^•^) as mentioned previously.

Various degrees of effect on myocardial cells have been observed using exogenous catalase to protect against free radical damage (Gross *et al*, 1986; Przyklenk and Kloner, 1986; Jeroudi *et al*, 1990). Actually, some reports demonstrated no protection at all *in vivo* (Bjorkman *et al*, 1991; Villani *et al*, 1992). These conflicting results were attributed to several limiting factors among which the short half-life and rapid clearance of the exogenously administered catalase were regarded as the main hurdles. Another factor is the poor endocytosis of exogenous catalase by cardiac myocytes (Chudej *et al*, 1990), which suggests a limited penetration of catalase into doxorubicin-treated cells and thereby limits the protective effect of catalase.

These limitations were circumvented by using transgenic mice overexpressing catalase. Several studies have been undertaken to investigate the susceptibility of these animals to free radical producing treatments (e.g. doxorubicin and IR). A 60-fold increase in catalase activity protected against the decrease in contractile force and morphological changes induced by hypoxia reoxygenation (H/R) (Chen *et al*, 1997). In another study, a high increase in catalase (60-fold) promoted the recovery of the contractile function of isolated hearts and to protect against creatine kinase CK efflux (Li *et al*, 1997).

A 60–100-fold increase in catalase expression protected against the increase in lipid peroxidation and plasma CPK in doxorubicin-treated transgenic mice, while lower or higher increases (15- and 200-fold, respectively) did not (James Kang *et al*, 1996). This study illustrates a bell-shape protective effect of catalase.

The present study was designed to increase the level of catalase alone while maintaining the endogenous levels of catalase aiming to overcome hydroxyl radical damage. Significant levels of catalase were produced in NeRCaMs at the third day of AdCat infection compared to mock-infected cells. However, the occurrence of cellular cytotoxicity limited the MOI of AdCat to 50 pfucell^−1^, which only produced a 3.5-fold increase in the activity of catalase in infected cells at the third day of infection compared to blank. This may explain why the 3.5-fold increase in the level of catalase did not protect against doxorubicin cardiotoxicity. Others showed that a nine-fold increase in catalase level in vascular endothelial cells (HUVECs) *in vitro* could protect against the damage induced by 0.5 but not by 1 mM H_2_O_2_ (Erzurum *et al*, 1993). In another study, a five-fold increase in catalase activity after lung gene transfer of catalase did not protect against ischemia perfusion-induced free radical in an isolated perfused lung model (Danel *et al*, 1998). Therefore, it is clear from the different studies that surplus amount of catalase should be achieved to counteract the burst of radicals produced by doxorubicin. The expression level of catalase produced by the present AdCat cannot be induced further since expression of the catalase gene is driven by the major late promoter of adenovirus 2, which is known to be among the strongest promoters available.

Doxorubicin alone decreased the viability of NeRCaMs in a concentration-dependent manner. This decrease was accompanied by an increase in the LDH release from treated cells. This is in line with previous reports, which showed that the free radicals produced by doxorubicin interact with the phospholipids of the cell membrane and produce membrane damage (Sterrenberg *et al*, 1984).

It is remarkable that when all the cells completely stopped beating, a fair fraction (10–16%) was still viable. This indicates that high energy demanding processes like beating stops before the complete death of cells. This probably resulted from a doxorubicin-induced reduction of cellular ATP levels (Cini Neri *et al*, 1991).

Recently, the beneficial effects of flavonoids have been shown in the treatment of different diseases (Wagner, 1985). Among them monoHER, in contrast to catalase, significantly protected against doxorubicin cytotoxic effects (Survival, LDH release and beating rate). The pronounced protection of monoHER was most probably caused by its site-specific action as a potent antioxidant and a metal ion chelator (Van Acker *et al*, 1996, 1998). 7-Monohydroxyethylrutoside may also be a good protector against membrane damage because it is one of the constituents of the flavonoid mixture Venorotun® that is used in the treatment of chronic venous insufficiency presumably via the stabilisation of collagen (Wagner, 1985). The combination of these properties makes monoHER a powerful protector against doxorubicin-induced cardiotoxicity.

In conclusion, the present study shows that a 3.5-fold increase in the level of catalase in cultured NeRCaMs was not enough to protect against doxorubicin-induced cardiotoxicity. In contrast, monoHER protected NeRCaMs against doxorubicin-induced membrane damage (as illustrated by the increase in the LDH release from treated cells) and decrease in viability of treated cells. 7- Monohydroxyethylrutoside also protected against the negative chronotropic action of doxorubicin on cardiac myocytes, which is in agreement with the cardioprotecting effects of monoHER described earlier (Van Acker SABE *et al*, 1997; Van Acker FAA *et al*, 2000).
